# C-phycocyanin alleviated cisplatin-induced oxidative stress and inflammation *via* gut microbiota—metabolites axis in mice

**DOI:** 10.3389/fnut.2022.996614

**Published:** 2022-09-20

**Authors:** Yubing Zhang, Lili Li, Song Qin, Jingyi Yuan, Xiaonan Xie, Fan Wang, Shanliang Hu, Yuetao Yi, Min Chen

**Affiliations:** ^1^College of Life Sciences, Yantai University, Yantai, China; ^2^Yantai Institute of Coastal Zone Research, Chinese Academy of Sciences, Yantai, China; ^3^Center for Ocean Mega-Science, Chinese Academy of Sciences, Qingdao, China; ^4^Center for Mitochondria and Healthy Aging, College of Life Sciences, Yantai University, Yantai, China; ^5^Department of Radiotherapy, Yantai Yuhuangding Hospital, Yantai, China

**Keywords:** C-phycocyanin, cisplatin, gut microbiota, metabolomics, inflammation, oxidative stress, FAHFAs, chemotherapy

## Abstract

C-phycocyanin is a natural protein extracted from *Spirulina platensis*. We aim to investigate the preventive effect of C-phycocyanin on cisplatin chemotherapy-induced oxidative damage and inflammation. The result showed that C-phycocyanin treatment reduced cisplatin-induced mortality and inflammation including decreased levels of serum IL6, kidney MCP1, and liver IL1β. Furthermore, C-phycocyanin also exerted antioxidant effects on mice, including increased GSH-Px, GGT, and GSH levels in the liver and increased CAT and SOD levels in the kidney. HepG2 cells experiments showed that C-phycocyanin exhibited none of the prevention effects on cisplatin injury. *Faecalibaculum* showed the greatest reduction among genera after cisplatin treatment, which was related to the enrichment of *Romboutsia* and *Lactobacillus* genera. C-phycocyanin treatment reduced the populations of harmful bacteria of *Enterococcus faecalis*, which was positively correlated with inflammation induced by cisplatin. C-phycocyanin increased the contents of 23-nordeoxycholic acid and β-muricholic acid. Moreover, C-phycocyanin increased amino acid-related metabolites, N_α_-acetyl-arginine and trimethyl-lysine contents, and decreased fatty acid esters of hydroxy fatty acids (FAHFAs) contents. In conclusion, C-phycocyanin inhibited inflammation *via* the 23-nordeoxycholic acid-*Enterococcus faecalis*-inflammation axis, and enhanced the antioxidant capacity of kidney *via Lactobacillus*-NRF2 pathway. C-phycocyanin alleviated cisplatin injury *via* the modulation of gut microbiota, especially *Lactobacillus* and *Enterococcus*, as well as regulation of metabolites, especially bile acid and FAHFAs, which highlight the effect of C-phycocyanin and provide a new strategy to prevent cisplatin injury.

**Graphical Abstract:**
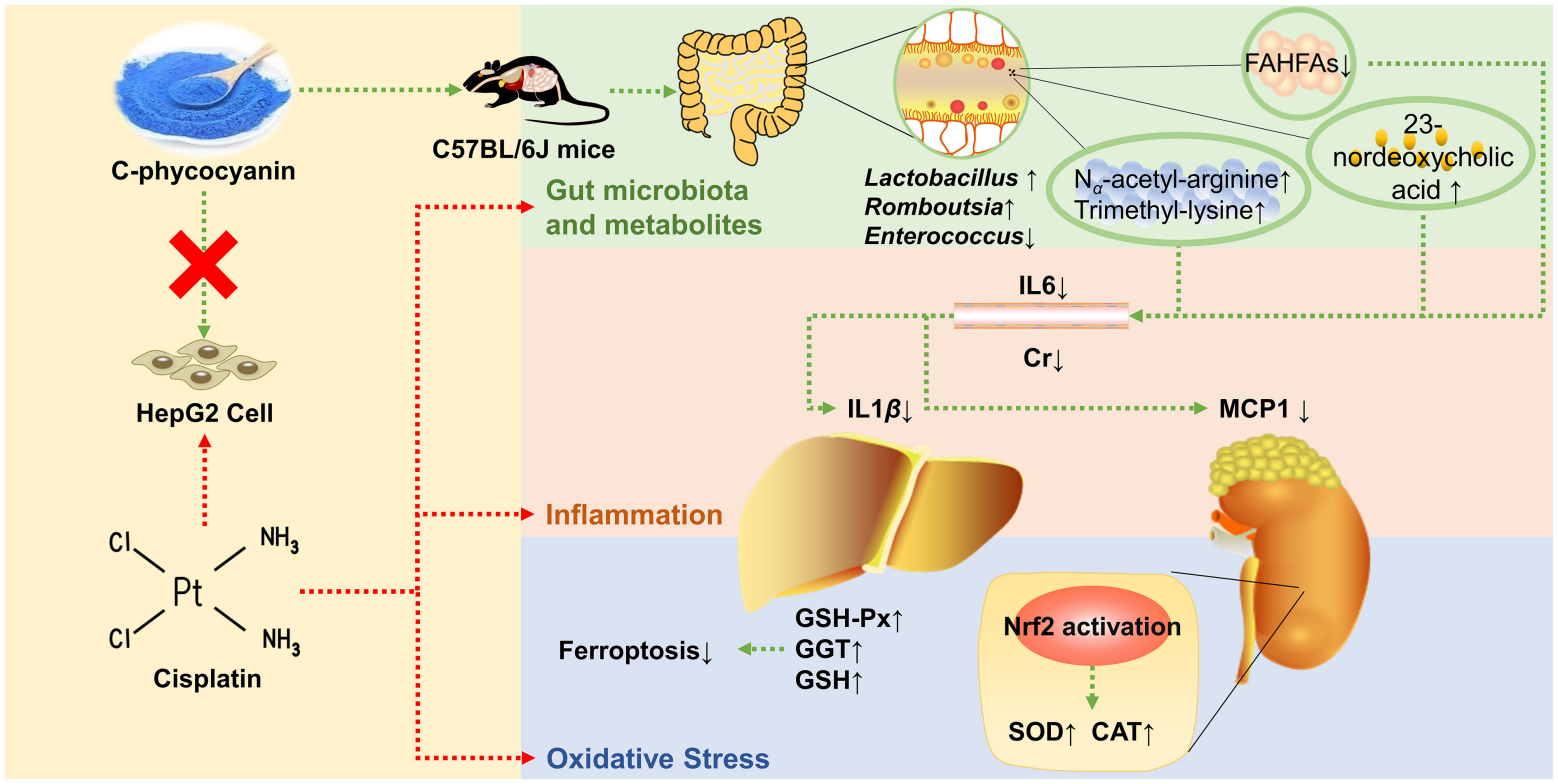


## Introduction

Cancer is a leading cause of death worldwide, accounting for nearly 10 million deaths in 2020, according to an analysis by the World Health Organization (WHO). Cisplatin-based chemotherapy is currently the main regimen for systemic treatment of advanced cancer (1), and cisplatin is a highly effective antineoplastic drug used for treating a myriad of solid tumors (2). Although cisplatin is useful in treating numerous tumors, it causes cell damage through oxidative stress and inflammation (3), especially in the kidney and liver. It has been reported that the hepatotoxicity induced by cisplatin is caused by the production of reactive oxygen species, the weakening of antioxidant defense system, and the cascade of the inflammatory response (4, 5). Acute kidney injury (AKI) is the most common side effect of cisplatin. At present, there is no specific strategy that can continuously reduce or prevent cisplatin-induced AKI (6). Therefore, it is urgent to develop a treatment to reduce toxicity and side effects.

C-phycocyanin derives from *Spirulina platensis*, which has been reported to protect the liver and kidney (7). However, due to its huge molecular weight, it is usually difficult to be absorbed by cells, so it is necessary to evaluate its biological activity *via in vitro* experiment. The latest *in vivo* experiments using non-degraded C-phycocyanin also showed anticancer and other activities (8). Although *in vitro* studies of C-phycocyanin injection have found its anticancer, antioxidant, and anti-inflammatory effects (9, 10), because C-phycocyanin belongs to macromolecular foodborne substances, it is concluded that the expected effect can be achieved only by injection *in vivo*, which has a great test in its safety. Therefore, oral administration has a higher value at present. But C-phycocyanin, as a protein, can be degraded by the host-produced digestive enzymes into polypeptide chains which is also a great difficulty that limits the revealing of the oral mechanism of C-phycocyanin. In addition to the digestive enzymes produced by the host, gut microbiota plays a vital role in the metabolites of C-phycocyanin. Oral C-phycocyanin showed a protective effect on ethanol-induced gastric ulcers in rats (11), but whether gut microbiota and their metabolites are involved in this effect is unclear. Gut microbiota involved in various diseases is gradually revealed. Gut microbiota has even become a biomarker for the treatment and diagnosis of some complex diseases, such as bipolar disorder (12), chronic spontaneous urticaria, and symptomatic dermographism (13). As summarized in our previous review, the impact of the gut microbiota on host health is mainly achieved through microbial metabolites (14).

Therefore, to explore the preventive mechanism of C-phycocyanin against cisplatin-induced damage, the cell activity of the digested C-phycocyanin will be first evaluated *via in vitro* experiment. The effects of C-phycocyanin improving cisplatin-induced chemotherapy injury through gut microbiota and its metabolites will be further clarified in mice.

## Materials and methods

### Animals and diets

C-phycocyanin (CAS number: 11016-15-2) was purchased from the Zhejiang Binmei Biotechnology Co., Ltd. (Zhejiang, China). Cisplatin (*cis*-diammineplatinum dichloride CAS number: 15663-27-1) was purchased from the Shanghai Macklin Biochemical Co., Ltd. (Shanghai, China). SPF C57BL/6J mice (6 weeks old, male) were obtained from the Jinan Pengyue Experimental Animal Technology Co., Ltd. (Shandong, China). All mice were acclimatized for 1 week before experiments. Mice were randomly divided into three groups with 10 mice per group. The group without cisplatin (i.p.), which was treated with phosphate-buffered saline (PBS), was named the NC group; the group with PBS intervention after cisplatin chemotherapy (2 weeks later, 10 mg/kg cisplatin in PBS buffer solution 0.2 mL was injected intraperitoneally (15, 16); the other groups were only injected with PBS 0.2 mL) treatment was named the CIS group; the group with C-phycocyanin intervention after chemotherapy was named the PC group. C-phycocyanin (5 mg/mL) (17, 18) in PBS or PBS alone was gavage administered to mice in the PC group (0.2 mL), CIS group (0.2 mL), and NC group (0.2 mL) for 3 weeks. The study was conducted according to the guidelines of the Declaration of Helsinki. The experiment was carried out in the Yantai Yuhuangding hospital and approved by the ethics committee of the Yantai Yuhuangding hospital (Approval NO. 404-2019). The *in vivo* experimental design process is shown in Table 1. The feces were collected in a sterilized single cage per mouse. The fresh feces were immediately frozen in liquid nitrogen. At the end of the experiment, after anesthesia with pentobarbital sodium 50 mg/kg, the mice were killed by the decapitation method. After the mice died, the liver and kidney were immediately collected, and the blood was washed off with PBS and immediately frozen in liquid nitrogen.

**Table 1:** The *in vivo* experimental design protocol.

**Day**	**NC group**	**CIS group**	**PC group**
−7		Mice were acclimatized for 1 week	
0–21	Daily gavage with PBS	Daily gavage with PBS + PC (50 mg/kg, 0.2 mL)	
14	PBS intraperitoneal injection	PBS + CIS intraperitoneal injection (10 mg/kg, 0.2 mL)	
19–20	Feces were collected for gut microbiota and microbiome analyses		
21	The mice were killed by cervical dislocation and the liver and kidney tissues were obtained		

NC Group, Normal control group; CIS Group, Cisplatin treated group; PC Group, Phycocyanin treated group; PBS, Phosphate buffer saline.

### *In vitro* experiment

HepG2 cell line was purchased from Procell Life Science and Technology Co., Ltd. (Hubei, China). C-phycocyanin and cisplatin were dissolved in Dulbecco's modified eagle medium (DMEM), respectively. C-phycocyanin solution was further digested with trypsin, and then centrifuged with a 10 kDa ultrafiltration centrifuge tube to obtain C-phycocyanin degradation product; then two solutions over 0.22 μm filter membrane were used to prepare sterile solutions: C-phycocyanin 5 mg/mL (consistent with the concentration of animal experiment) and cisplatin 0.025 mg/mL [1 mg/mL cisplatin solution was obtained at a concentration of 3,333.2 μmol/L (19). The 48-h IC50 of HepG2 cells is 16 μmol/L (20)]. The sterile solutions were diluted with high sugar DMEM and fetal bovine serum (FBS) to make a cell culture medium. NC cell group FBS/DMEM = 1/9 (v/v), NPC cell group PC/FBS/DMEM = 1/1/8 (v/v/v), CIS cell group CIS/FBS/DMEM = 1/1/8 (v/v/v), and PC cell group PC/CIS/FBS/DMEM = 1/1/1/7 (v/v/v/v). Photos were taken using Leica DMi8 automated imaging system (Leica, Germany). Cell viability was detected by an MTT kit from Beijing Solarbio Science and Technology Co., Ltd (Beijing, China), CN (catalog number): M1020. The *in vitro* experimental design process is shown in Table 2.

**Table 2:** The *in vitro* experimental design protocol.

**Group**	**NC cell group**	**NPC cell group**	**CIS cell group**	**PC cell group**
Intervention medium	FBS/DMEM 1/9 (v/v)	PC/FBS/DMEM 1/1/8 (v/v/v)	CIS/FBS/DMEM 1/1/8 (v/v/v)	PC/CIS/FBS/DMEM 1/1/1/7 (v/v/v/v)
Time	Treatment method			
−24 h	HepG2 cells were seeded in 6-well plates with an inoculation volume of 3 mL and cell density is 3.5 × 10^4^ cell/mL.			
0 h	Replace the complete medium with the corresponding intervention medium and take photos.			
24 h	Take pictures at the same place as the first photographing position.			
48 h	Take pictures at the same place as the first photographing position.			

### Enzyme-linked immunosorbent assay (ELISA) analysis

The levels of serum creatinine (Cr) CN: ML037726, kidney superoxide dismutase (SOD) CN: ML643059, catalase (CAT) CN: ML037752, NF-E2-related factor 2 (NRF2) CN: ML037744, liver glutathione peroxidase (GSH-Px) CN: ML037757, γ-glutamyl transferase (GGT) CN: ML994533, and glutathione (GSH) CN: ML063305 were determined according to the manufacturer's instructions using ELISA kits obtained from Mlbio Company (Shanghai, China). The levels of serum interleukin 6 (IL6) CN: M6000B, kidney monocyte chemoattractant protein 1 (MCP1) CN: MJE00B, and liver interleukin 1β (IL1β) using ELISA kits were bought from R&D Systems CN: MLB00C (Minneapolis, USA). The nuclear protein of renal tissue was extracted using the nuclear and cytoplasmic protein extraction kit obtained from Labgic Technology Co., Ltd. (Beijing, China).

### Microbiota analysis

Microbiota samples were extracted from feces using a QIAamp DNA stool kit (QIAGEN Inc., Germantown, MD, USA). The variable regions V3-V4 of the 16S rRNA genes were amplified using the primers 515F (′GTGCCAGCMGCCGCGGTAA′) and 806R (′GGACTACHVGGGTWTCTAAT′). The purified amplicons were analyzed using paired-end sequencing on the Illumina NovaSeq system (San Diego, CA, USA) by Novogene Co., Ltd. (Beijing, China). The analysis was conducted according to our previous work (21). The clean sequence data of the mouse fecal microbiota had been deposited in NCBI Sequence Read Archive (SRA) under the accession number PRJNA777291.

### Metabolome analysis

The metabolome was analyzed using UHPLC-MS/MS performed on a Vanquish UHPLC (Thermo Fisher, Germany) coupled with a Q Exactive HF-X mass spectrometer (Thermo Fisher, Germany) by Novogene Co., Ltd. (Beijing, China). Samples were separated using a Hypesil Gold column (100 × 2.1 mm, 1.9 μm) with a flow rate of 0.2 mL/min. The corresponding analysis parameters were carried out according to the previous method (22).

### Statistical analysis

Data were analyzed by using IBM SPSS Statistics version 26.0 (International Business Machines Corporation, USA). One-way ANOVA was used to evaluate the difference between two or three groups, and *p* < 0.05 was considered to be significantly different. The correlation heat map was made by using Origin 2022 (Origin Lab Corporation, USA).

## Results

### C-phycocyanin improves weight loss and acute death

**Figure 1:**
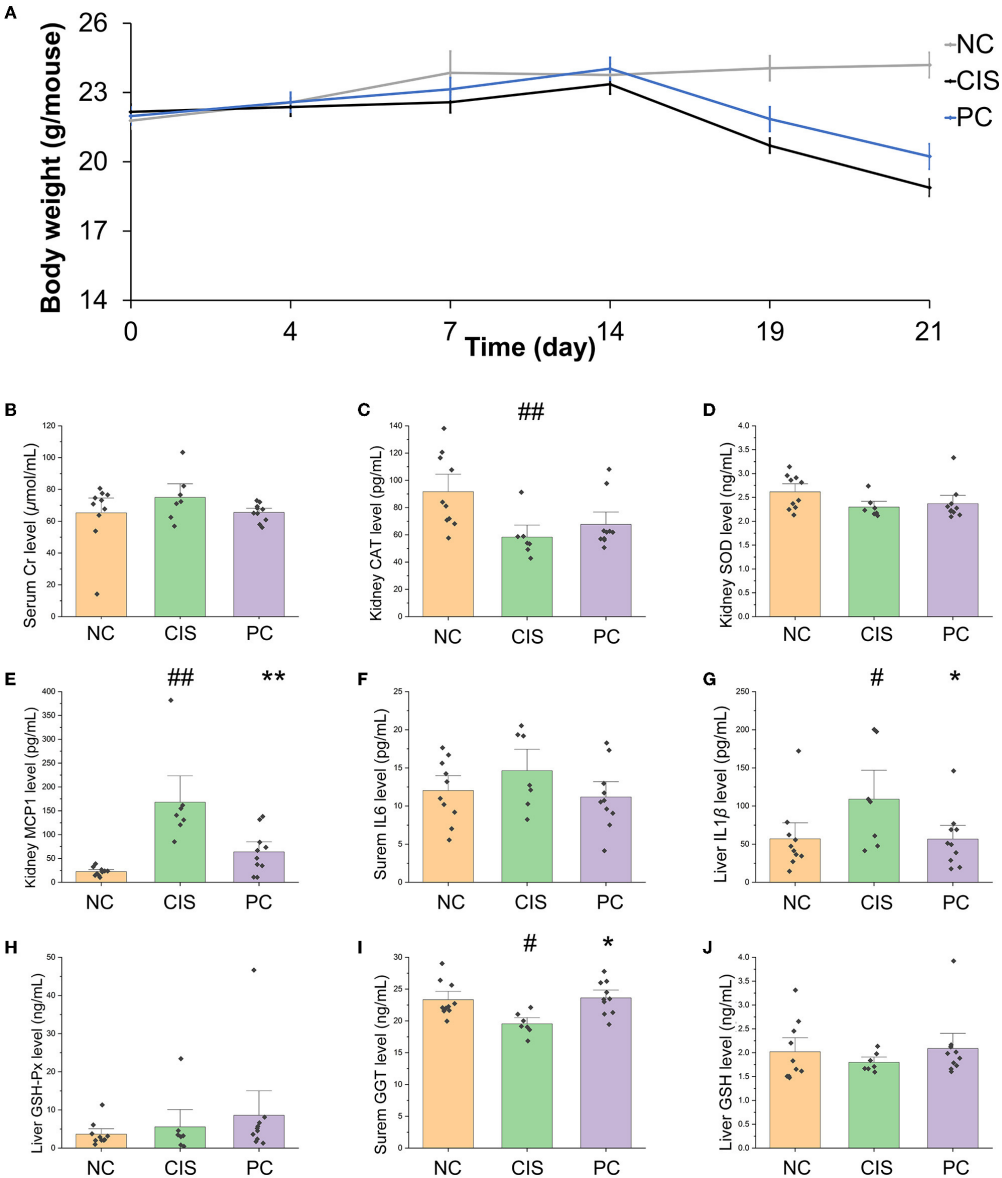
Body weight and biochemical parameters of blood, kidney, and liver in mice. **(A)** Before cisplatin intervention, there were no significant differences in body weight among the three groups (*p* > 0.05). Cisplatin intervention significantly decreased body weight compared with the NC group on the 19th and 21st day (*p* < 0.01). **(B)** serum Cr level; **(C)** kidney CAT level; **(D)** kidney SOD level; **(E)** kidney MCP1 level; **(F)** serum IL6 level; **(G)** liver IL1β level; **(H)** liver GSH-Px level; **(I)** liver GGT level; **(J)** liver GSH level. The marker on PC column indicates the significant difference of PC vs. CIS (*p < 0.05, **p < 0.01); the marker on CIS column indicates the significant difference of CIS vs. NC (#*p* < 0.05, ##*p* < 0.01). NC, normal control; CIS, cisplatin; PC, cisplatin with C-phycocyanin.

There were no significant differences in average body weight among the NC, CIS, and PC groups in the first 2 weeks. After the cisplatin treatment, the body weight of the CIS group and the PC group decreased significantly compared with the NC group in the last week (Figure 1A). The PC group showed less weight loss compared with the CIS group. After cisplatin treatment, the 7-day survival rate in the CIS group was 70% (one mouse died before fecal samples collect and two mice died before euthanasia), whereas the 7-day survival rate in the PC group was 100%, which indicated that C-phycocyanin improved the weight loss and enhanced survival rate caused by cisplatin chemotherapy.

### C-phycocyanin prevented oxidative damage to liver and kidney

The intervention of C-phycocyanin reduced the levels of serum Cr and kidney MCP1, and increased the levels of kidney CAT and SOD, which suggested that it improved the oxidative damage to kidney (Figures 1B–E). C-phycocyanin treatment reduced the level of serum IL6, which indicated its anti-inflammation effects (Figure 1F). The intervention of C-phycocyanin has reduced the level of liver IL1β, and increased liver GSH-Px, GGT and GSH levels, which suggested its prevention role against liver inflammation and oxidative damage (Figures 1G-J).

### The preventive effect of C-phycocyanin on cisplatin chemotherapy needs to be realized through intestinal digestion

**Figure 2:**
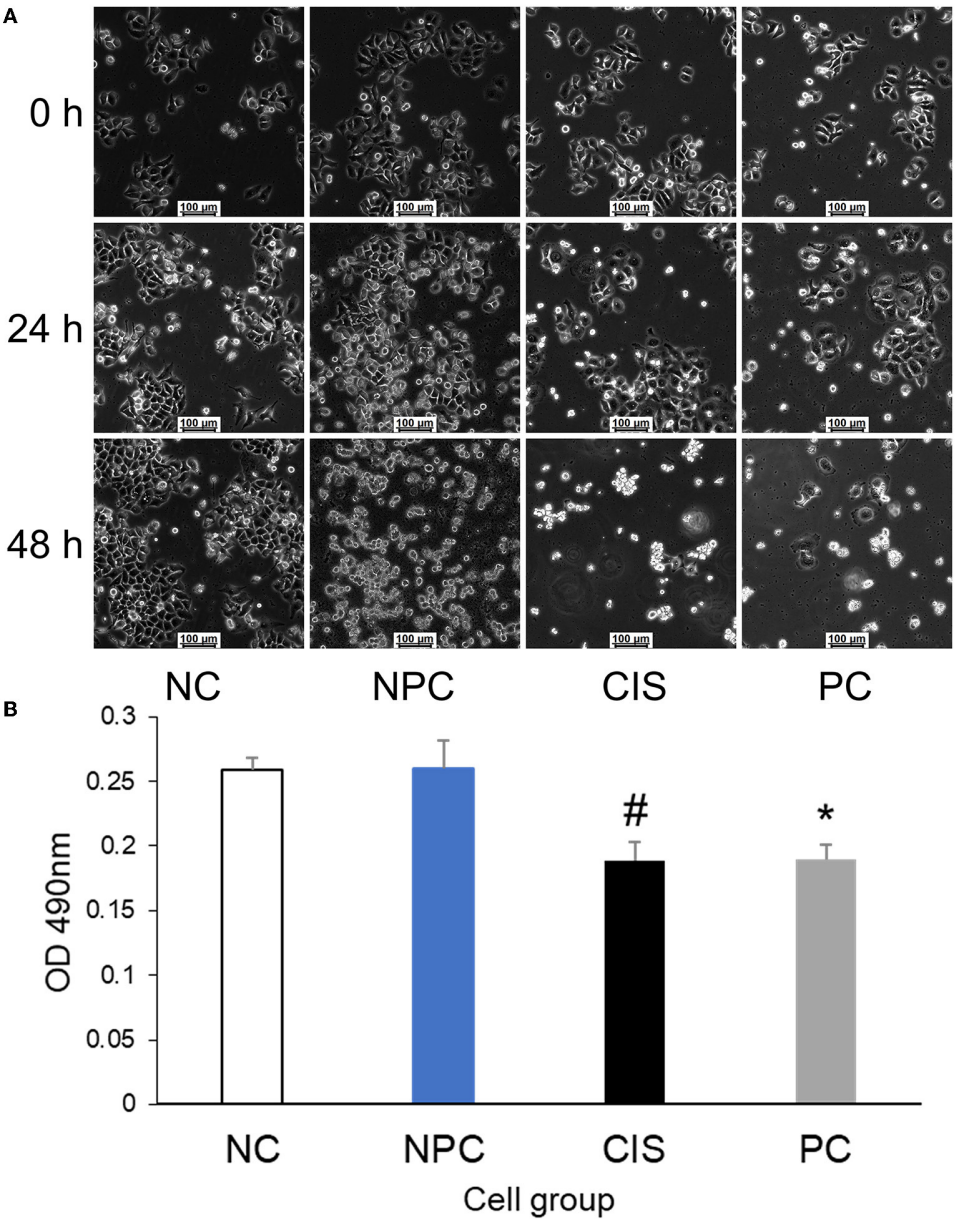
Effects of C-phycocyanin on the viability of HepG2 cells. **(A)** Cell morphology of HepG2 cells at the same site for 48 h; **(B)** MTT test of HepG2 cells. The CIS group compared with the NC and NPC groups (#*p* < 0.01); the PC group compared with the NC and NPC groups (*p < 0.05). NC, normal control; NPC, normal control with C-phycocyanin; CIS, cisplatin; PC, cisplatin with C-phycocyanin.

To determine whether C-phycocyanin itself has a prebiotic effect on cells, the results of fixed-point photography of cell morphology at 0 h, 24 h, and 48 h showed that the cell growth rate of the NC and NPC groups was similar, whereas the cell growth rate of the CIS and the PC groups were slower than NC and the NPC groups during the first 24 h and there was serious apoptosis at 48 h (Figure 2A). Then, we used HepG2 cells for the MTT experiment *in vitro* (Figure 2B). The result showed that C-phycocyanin addition *in vitro* did not improve the cytotoxicity caused by cisplatin (*p* > 0.05). It indicated that the preventive effect of C-phycocyanin on cisplatin toxicity is indirect.

### C-phycocyanin improved the microbiota disorder

**Figure 3:**
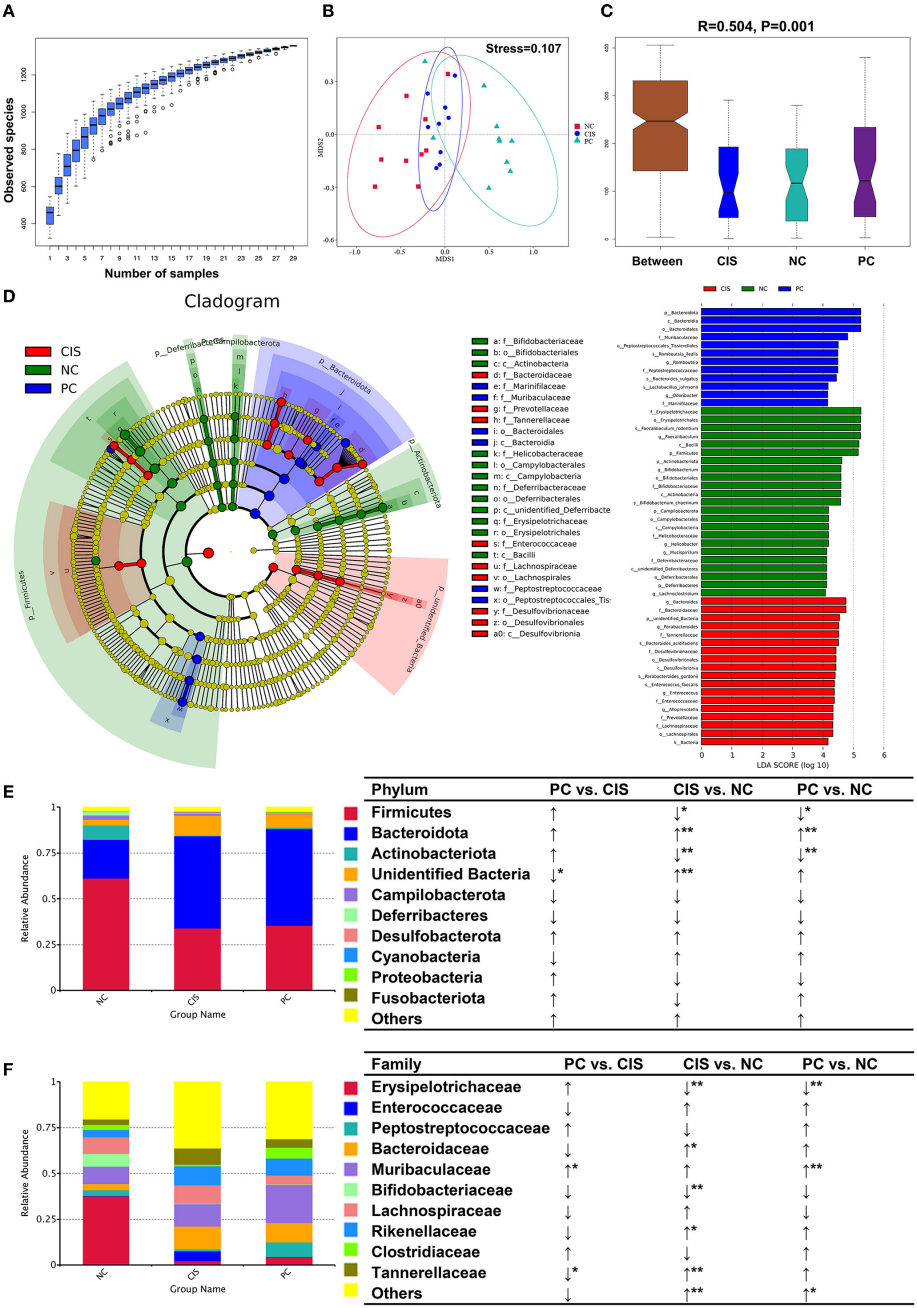
Gut microbiota analysis. **(A)** Species accumulation boxplot of all samples; **(B)** non-metric dimensional scaling (NMDS) plots; **(C)** analysis of similarities (ANOSIM) showed the significance of separation among the NC, CIS, and PC groups; **(D)** Linear discriminant analysis Effect Size (LEfSe) analysis (the filter value of LDA score is 4); **(E)** relative abundance of the top 10 phyla; **(F)** relative abundance of the top 10 families. *n* = 10 for the PC and NC groups, *n* = 9 for the CIS group (one mouse in the CIS group died before collecting feces). NC, normal control; CIS, cisplatin; PC, cisplatin with C-phycocyanin; *p < 0.05, **p < 0.01; ↑, upregulation; ↓, downregulation.

The whole gut microbiota analysis exhibited that there were significant differences in microbiota composition among the NC, CIS, and PC groups (Figures 3A–C). At the phylum level, C-phycocyanin increased the abundance of Firmicutes, Actinobacteriota, Proteobacteria, and Fusobacteriotacom compared with the CIS group. C-phycocyanin decreased the abundance of Cyanobacteria compared with the CIS group (Figures 3D,E). At the family level, C-phycocyanin increased the abundance of Erysipelotrichaceae, Peptostreptococcaceae, Muribaculaceae, and Clostridiaceae compared with the CIS group. C-phycocyanin decreased the abundance of Enterococcaceae, Bacteroidaceae, Bifidobacteriaceae, Lachnospiraceae, Rikenellaceae, and Tannerellaceae compared with the CIS group (Figure 3F).

### The core bacteria affected by C-phycocyanin and cisplatin are *the Lactobacillus* genus and *Enterococcus faecalis*

**Figure 4:**
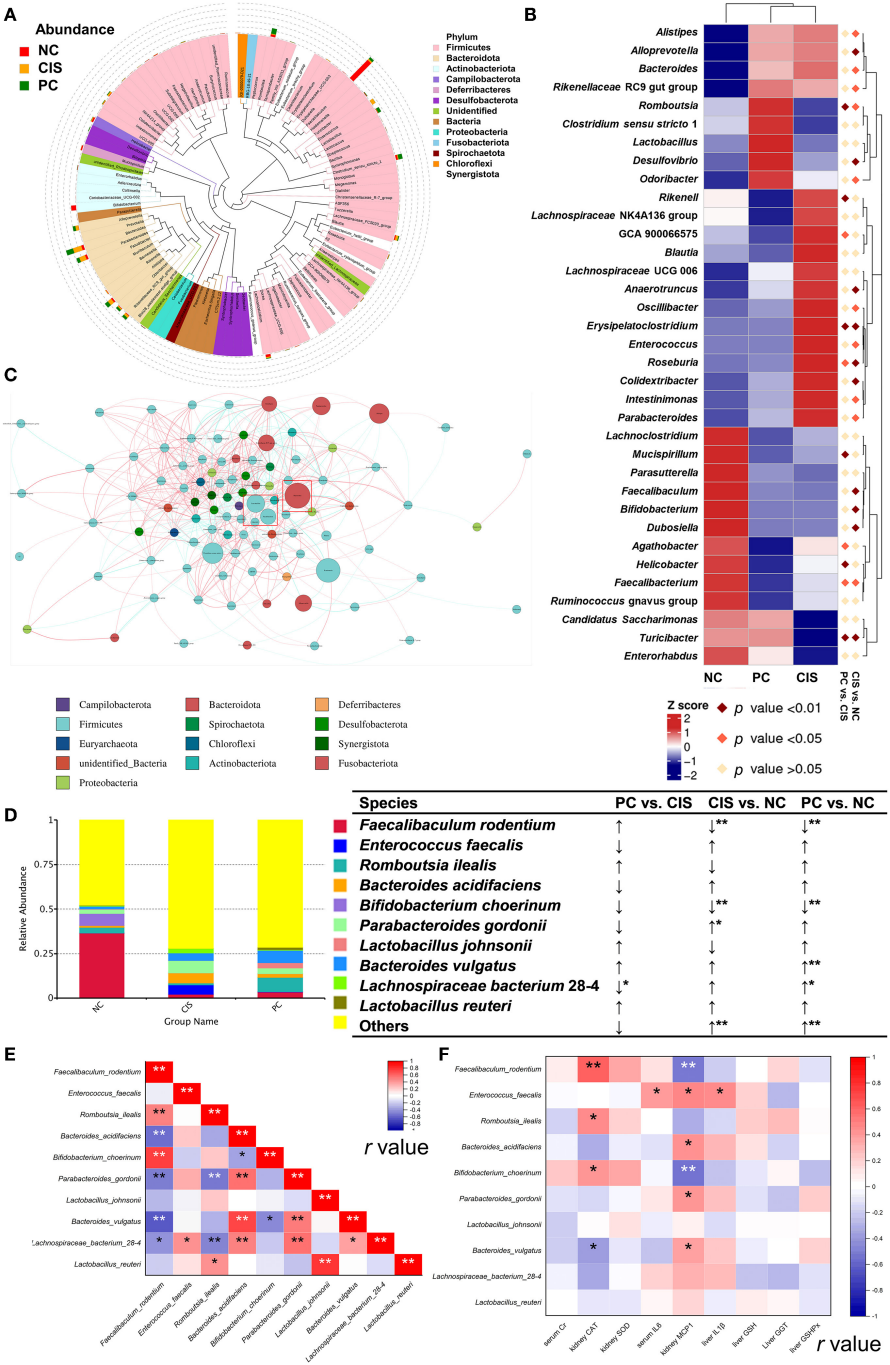
Gut microbiota analysis at genus level. **(A)** The top 100 genera evolutionary tree; **(B)** heatmap of the top 35 genera; **(C)** genus network analysis in PC group, genera in the red box with blue circle were *Lactobacillus* and *Faecalibaculum*, and genus in the red box with a red circle was *Bacteroides*; **(D)** relative abundance of the top 10 species; **(E)** Spearman correlation analysis among the top 10 species; **(F)** Spearman correlation analysis between abundances of species and levels of biochemical parameters. *n* = 10 for the PC and NC groups, *n* = 9 for the CIS group (one mouse in the CIS group died before collecting feces); NC, normal control; CIS, cisplatin; PC, cisplatin with C-phycocyanin;↑, upregulation; ↓, downregulation; *, *p* < 0.05; **, *p* < 0.01.

We focused our analysis on the genus and species levels, C-phycocyanin increased the abundance of *Faecalibaculum, Romboutsia, Clostridium sensu stricto* 1, and *Lactobacillus* compared with the CIS group. C-phycocyanin decreased the abundance of *Enterococcus Bacteroides* and *Parabacteroides* compared with the CIS group (Figure 4B). *Lactobacillus* and *Faecalibaculum* showed higher abundance than most of the other genera and are located in the center, which indicates the complex interactions with the other taxa (Figure 4C). However, the intervention of C-phycocyanin increased the abundance of *Lactobacillus* and *Faecalibaculum* and decreased *Bacteroides* (Figure 4B). At the species level, C-phycocyanin increased the abundance of *Faecalibaculum rodentium, Romboutsia ilealis*, and *Lactobacillus johnsonii* compared with the CIS group (Figure 4D). C-phycocyanin decreased the abundance of *Enterococcus faecalis, Bacteroides acidifaciens, Parabacteroides gordonii*, and *Lachnospiraceae bacterium* 28–4 compared with the CIS group (Figure 4D). *Lactobacillus johnsonii* was positively correlated with *Lactobacillus reuteri* significantly, *Lactobacillus reuteri* was positively correlated with *Romboutsia ilealis* significantly, and *Romboutsia ilealis* was positively correlated with *Faecalibaculum rodentium* (Figure 4E). *Enterococcus faecalis* was positively correlated with three inflammatory indexes significantly (Figure 4F).

### The intervention of C-phycocyanin changed the composition of gut metabolites

**Figure 5:**
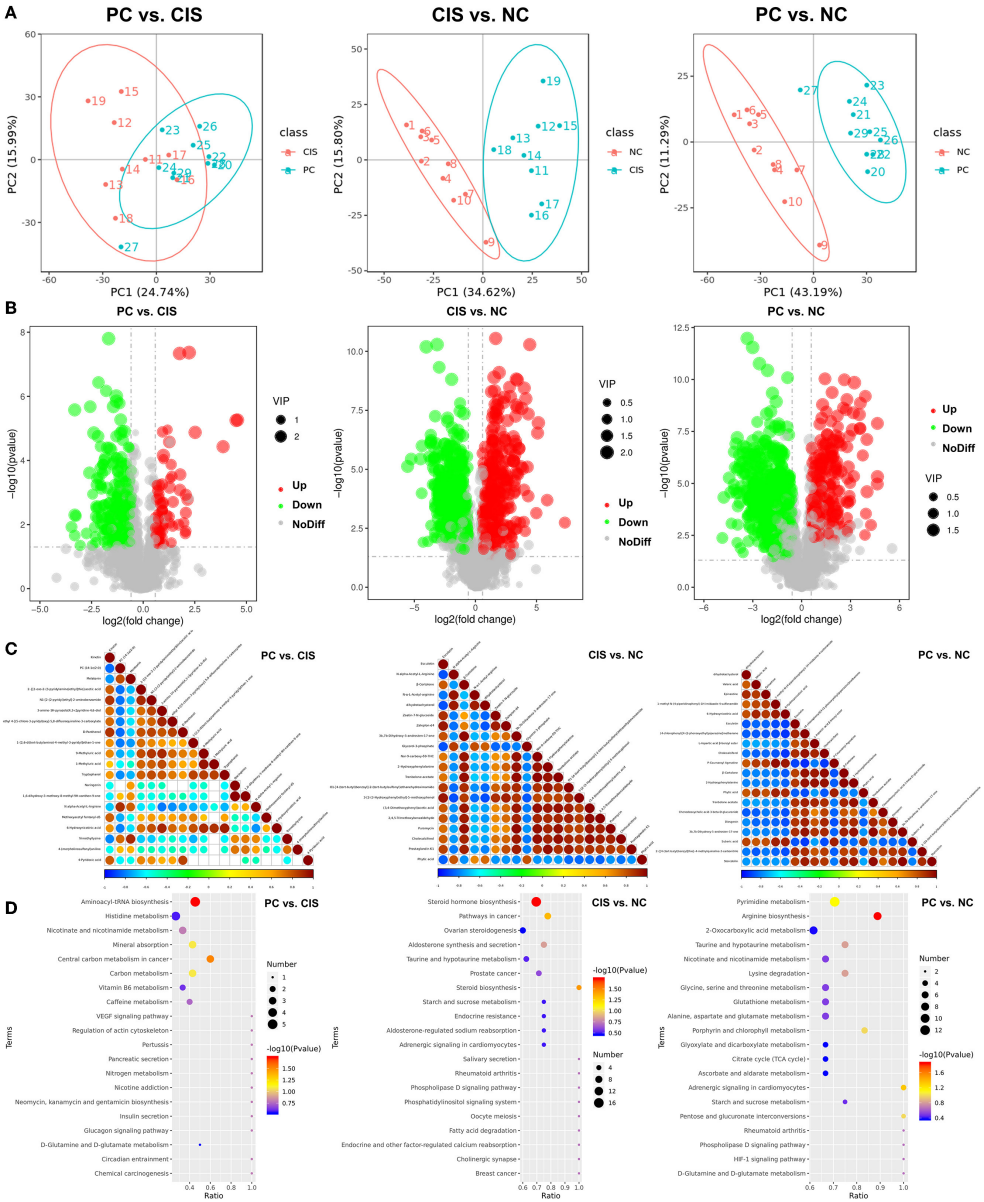
Metabolomics analysis in feces. **(A)** Principal components analysis (PCA) of all metabolic pathways in the three groups; **(B)** volcano map of differential metabolites in the three groups (Up: significantly increase *p* < 0.05; Down: significantly decrease *p* < 0.05; NoDiff: no significant difference *p* > 0.05); **(C)** the top 20 differential metabolite correlation plots in the three groups; **(D)** KEGG analysis of differential metabolites in the three groups. *n* = 10 for the PC and CIS groups, *n* = 9 for the CIS group (one mouse in the CIS group died before collecting feces). Z-score value represents the normalized abundance. VIP value represents the correlation coefficient. NC, normal control; CIS, cisplatin; PC, cisplatin with C-phycocyanin.

PCA analysis showed that there were significant differences in metabolites among the three groups (Figure 5A). The numbers of the downregulated microbial metabolites were larger than the upregulated metabolites after C-phycocyanin treatment compared with the CIS group (Figure 5B). The addition of C-phycocyanin significantly increased the amino acid-related metabolites, such as N_α_-acetyl-L-arginine and trimethyl-lysine (Figure 5C). N_α_-acetyl-L-arginine is an N-acetyl-L-amino acid that is L-arginine in which one of the hydrogens attached to the nitrogen is replaced by an acetyl group. It has a role as a host metabolite. It is a conjugate acid of N_α_-acetyl-L-arginine. Kyoto Encyclopedia of Genes and Genomes (KEGG) analysis showed that the metabolic pathway significantly changed by C-phycocyanin like aminoacyl-tRNA biosynthesis and central carbon metabolism in cancer is based on the pathway of amino acids and their metabolites (Figure 5D). KEGG enrichment analysis showed that the significantly different metabolic pathways between PC and CIS were aminoacyl-tRNA biosynthesis and central carbon metabolism in cancer, the significantly different metabolic pathway between CIS and NC was steroid hormone biosynthesis, and the significantly different metabolic pathway between PC and NC was arginine biosynthesis (Figure 5E).

### Bile acid metabolism is the main way for cisplatin injury and C-phycocyanin to prevent cisplatin injury

**Figure 6:**
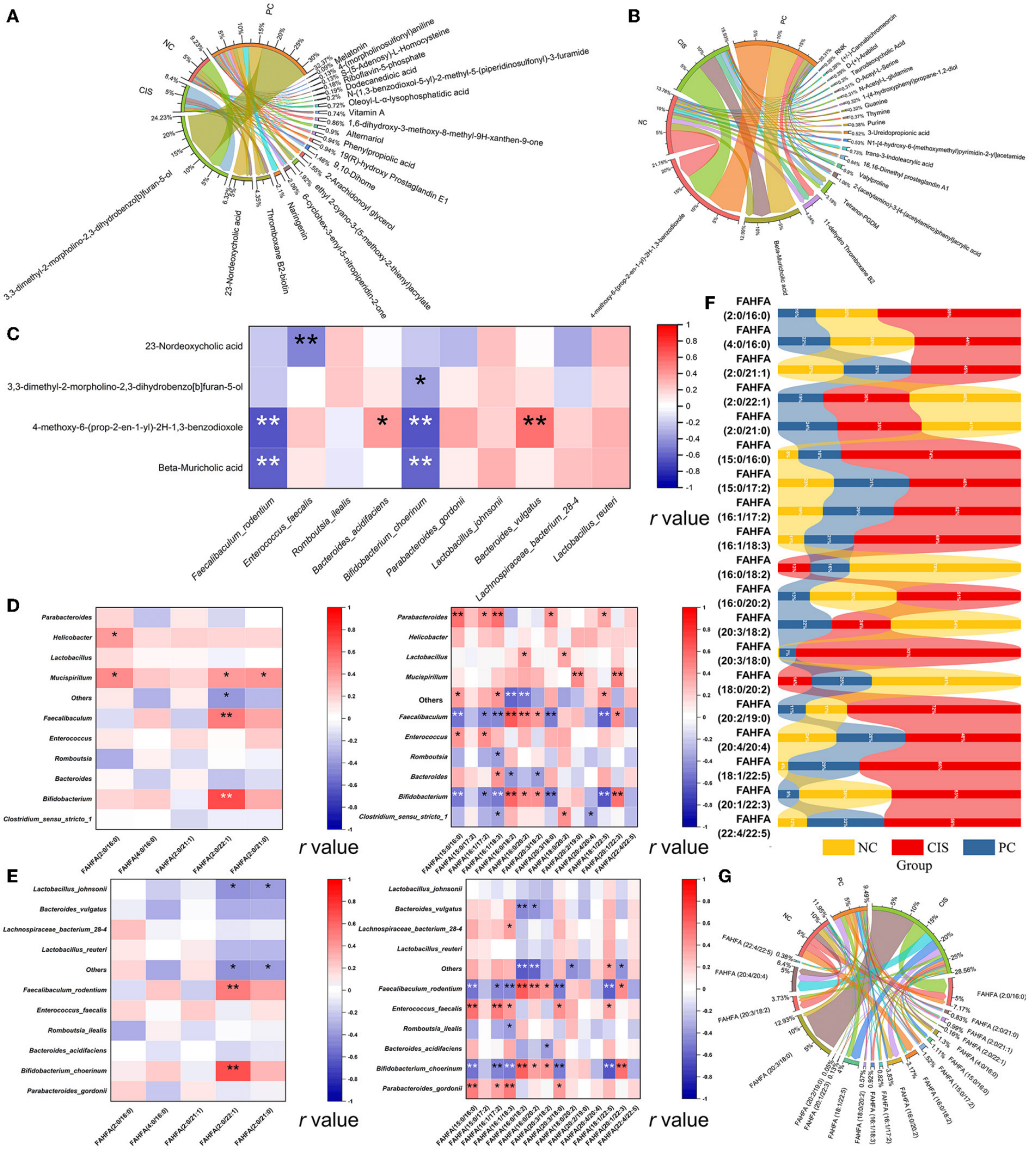
Bile acid-related metabolites and FAHFAs levels in feces. **(A)** Chord diagram of metabolites which was significantly regulated by C-phycocyanin compared with the CIS group; **(B)** chord diagram of metabolites which was significantly regulated by cisplatin compared with the NC group; **(C)** Spearman correlation analysis between bile acid metabolites and the top 10 species abundance; **(D)** Spearman correlation analysis between FAHFAs contents and the top 10 genera abundances; **(E)** Spearman correlation analysis between FAHFAs contents and the top 10 species abundances; **(F)** strip chart of relative content of all FAHFAs; **(G)** chord diagram between each group and FAHFAs. NC, normal control; CIS, cisplatin; PC, cisplatin with C-phycocyanin; *, *p* < 0.05; **p < 0.01.

To find the main metabolites of C-phycocyanin and cisplatin acting on the intestine, we screened all metabolites mainly affected by C-phycocyanin (PC vs. CIS, *p* < 0.01; CIS vs. NC, *p* > 0.05; PC vs. NC, *p* < 0.01) and metabolites mainly affected by cisplatin (PC vs. CIS, *p* > 0.05; CIS vs. NC, *p* < 0.01; PC vs. NC, *p* < 0.01). A total of 21 metabolites were screened by C-phycocyanin (Figure 6A); a total of 187 metabolites were screened by cisplatin, considering their content level, we only listed the 20 metabolites with the highest content (Figure 6B). The intervention of C-phycocyanin mainly increased the contents of 3,3-dimethyl-2-morpholino-2,3-dihydrobenzo[b]furan-5-ol and 23-nordeoxycholic acid. Cisplatin intervention mainly increased the contents of 4-methoxy-6-(prop-2-en-1-yl)-2h-1,3-benzodioxole and β-muricholic acid. Spearman correlation analysis showed that 23-nordeoxycholic acid was significantly negatively correlated with *Enterococcus faecalis* (Figure 6C).

### C-phycocyanin reduced the level of gut metabolite fatty acid esters of hydroxy fatty acids (FAHFAs)

Considering that fatty acid metabolites are closely related to bile acids, we noticed a new fatty acid complex FAHFAs. We classified all the compared FAHFAs according to the properties of their fatty acid chain. The fatty acid is short-chain fatty acids (SCFAs, chain length of one to six carbon atoms) called SCFAHFAs, and the fatty acid is short-chain fatty acids (LCFAs, chain length 6 carbon atoms) called LCFAHFAs. More correlation between FAHFA (2:0 / 22:1) and gut microbes at genera and species levels in SCFAHFAs implied that their contents were most likely regulated by gut microbes (Figures 6D,E). In the correlation analysis with LCFAHFAs, two strains, *Faecalibaculum rodentium* and *Bifidobacterium choerinum*, exhibited a complex correlation with LCFAHFAs and the two bacteria were the same in the trend of significance, implying the possibility of synergistic effects between the two strains on the metabolism of FAHFAs (Figure 6E). While, *Enterococcus faecalis* showed a positive correlation with five LCFAHFAs (Figure 6E). On the whole trend, C-phycocyanin reduced the increase of FAHFAs family members caused by cisplatin (Figure 6F). FAHFA (2:0/16:0) was the most abundant SCFAHFAs and FAHFA (20:3/18:0) was the most abundant LCFAHFAs in the match to, while the highest abundances of these two substances were in the CIS group (Figure 6G). Although the strip chart shows that FAHFA (2:0/22:1), FAHFA (2:0/21:0), FAHFA (16:0/18:2), FAHFA (20:3/18:2), and FAHFA (18:0/20:2) have higher abundances in the NC group, the contents of these five FAHFAs are in low levels and also not among the highest abundances of FAHFAs (Figures 6F,G).

### The intervention of C-phycocyanin activates the NF-E2-related factor 2 (NRF2) pathway in renal tissue

**Figure 7:**
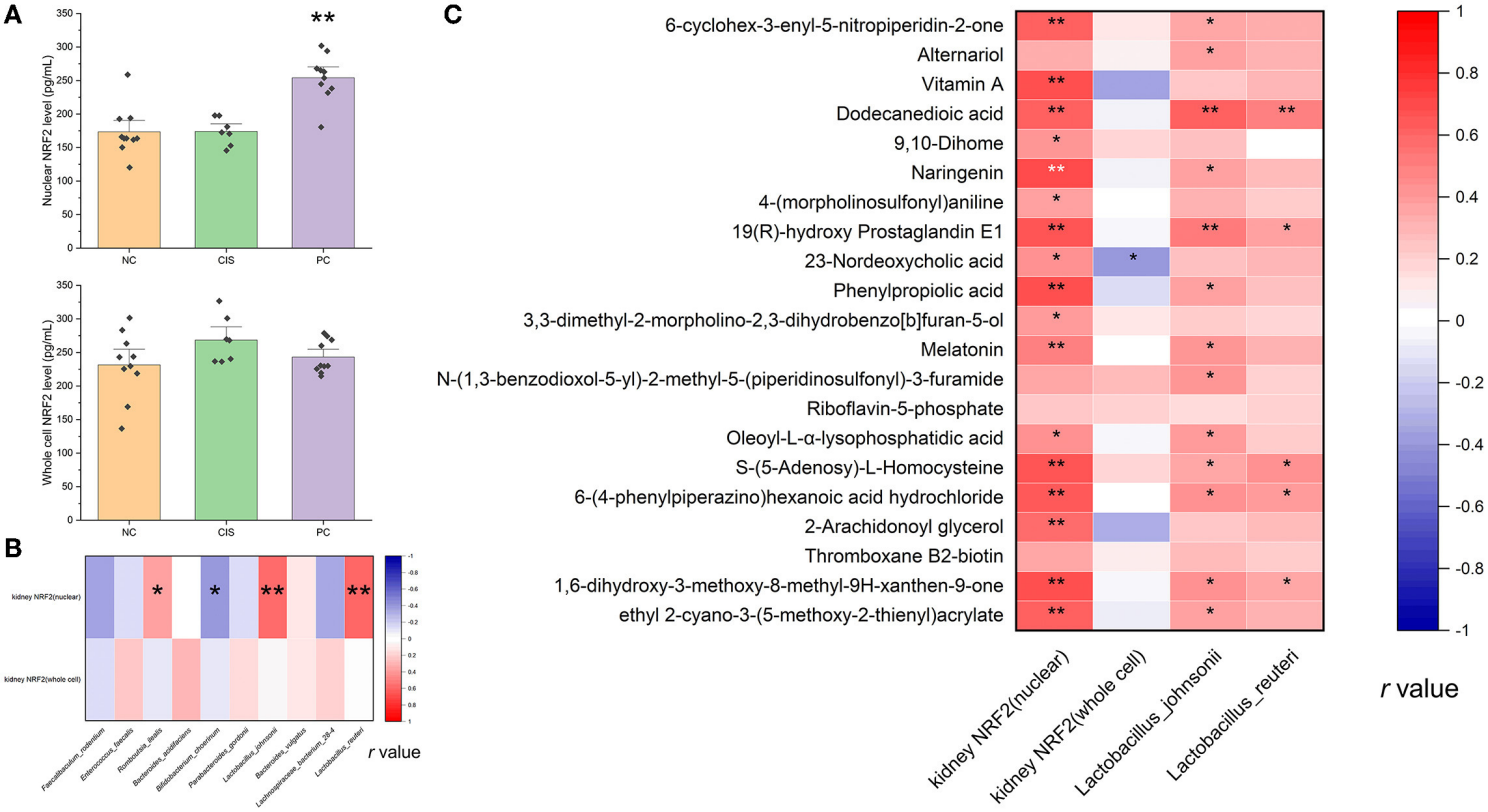
NRF2 protein level in renal tissue. **(A)** Nuclear and whole cell NRF2 protein level in kidney tissue, the marker on PC column indicates the significant difference of PC vs. CIS (**p < 0.01); **(B)** Spearman correlation analysis between NRF2 levels and the top 10 species abundances (*p < 0.05, **p < 0.01); **(C)** Spearman correlation analysis between NRF2 levels and all differential metabolites upregulated by C-phycocyanin compared with the CIS group (*p < 0.05, **p < 0.01). *n* = 10 for the PC and NC groups, *n* = 7 for the CIS group (three mice in the CIS group died before euthanasia). NC, normal control; CIS, cisplatin; PC, cisplatin with C-phycocyanin.

NRF2 is an important protein involved in oxidative stress response (23). NRF2 has to be activated to act as an antioxidant function. Activated NRF2 induces and regulates the expression of a series of downstream antioxidant factors in the nuclear. Therefore, we measured NRF2 levels in the kidney (Figure 7A). ELISA of nuclear NRF2 in renal tissue showed that the level of nuclear NRF2 protein in the PC group was significantly higher than that in the NC and CIS groups (*p* < 0.01), meanwhile, there was no significant difference in the level of the whole cell NRF2 protein among the three groups (*p* > 0.05). The activation of NRF2 was positively correlated with the genus of *Lactobacillus*. It was found that C-phycocyanin-related metabolites were mainly positively correlated with the contents of *Lactobacillus* and nuclear NRF2 levels (Figure 7B). We screened all metabolites due to C-phycocyanin intake (PC vs. CIS, *p* < 0.01; CIS vs. NC, *p* < 0.01; PC vs. NC, *p* > 0.05), and analyzed the correlation between these metabolites and the contents of *Lactobacillus* and NRF2 levels (Figure 7C).

## Discussion

C-phycocyanin has shown a preventive effect against weight loss and acute death caused by cisplatin, which means that intragastric PC can improve chemotherapy injury in mice. To verify the specific influencing factors, we detected nine biomarkers of inflammation and oxidative stress. Serum Cr is often used to evaluate kidney injury and is also an important predictor of cisplatin-associated AKI risk (24), and serum Cr decreased in our results, which means that the renal function of mice was improved. IL6, IL1β, and MCP1 are widely used as an indicator of the degree of inflammation in the body and its tissues (25). In our results, the levels of serum IL6 and liver MCP1, as well as kidney IL1β decreased after C-phycocyanin treatment, which means that C-phycocyanin can reduce the inflammation of the whole body, especially in the liver and kidney. There is a natural defense system against oxidative damage in the body, in which antioxidant enzymes are an important part. Antioxidant enzymes are mainly divided into three categories: SOD, CAT, and GSH-Px (26), the intervention of C-phycocyanin makes hepatocytes reduce oxidative damage through the GSH pathway, and renal cells reduce oxidative damage by increasing SOD and CAT levels.

SOD and CAT are mainly regulated by the NRF2 pathway. NRF2 bounds to the negative regulatory protein Kelch-like ECH-associated protein 1 (Keap1) in the cytoplasm, which interacts with NRF2 and acts as an adaptor protein to stabilize the normal condition of NRF2 (27). When oxidative stress occurs, Keap1 detects oxidative stress through the binding of redox-sensitive cysteine residues, such as cys151, cys273, and cys288, as well as releases NRF2 from Keap1 (28). When NRF2 is transferred to the nucleus, it binds to antioxidant response elements (AREs), which induces the transcription of genes related to cellular oxidoreductases, such as CAT and SOD (29). Therefore, we measured the NRF2 levels in the nucleus and in the whole cells of renal tissue. Compared with the CIS group, C-phycocyanin-activated NRF2 level in the nucleus of renal tissue was significantly increased, whereas there was no significant difference in whole cells between the CIS and PC groups. In our results, the levels of CAT and SOD in renal tissue increased in the C-phycocyanin group compared with the CIS group. Although the improvement of CAT and SOD levels is not significant, we believe that this is caused by a lack of the most serious individual data caused by three deaths in the CIS group before euthanasia.

GGT is an enzyme located on the surface of the cell membrane, which is involved in the maintenance of GSH metabolism and redox homeostasis. It has been found that high GGT expression is related to the resistance of cells to chemotherapy (30). GSH-Px, similar to SOD and CAT, is a key enzyme for removing peroxide in the biological system, but its antioxidant effect shows a dependence on GSH (31). In our results, the GSH-based antioxidant system of the liver was comprehensively elevated after the intervention of C-phycocyanin. Cisplatin treatment (10 mg/mL same dose as us) has been found to cause Ferroptosis (32), and cisplatin-induced depletion of GSH and inactivation of GSH-Px play a central role in the mechanism of Ferroptosis (33). This means that different from the antioxidant effect on kidney, the antioxidant effect of C-phycocyanin on liver might be resisting Ferroptosis by the GSH pathway.

To find out the path through which the anti-inflammatory and antioxidant effects of C-phycocyanin, we simulated the digestion of C-phycocyanin in the intestine *in vitro*, and intervened in the digested C-phycocyanin polypeptide in HepG2 cells. It was found that the digested C-phycocyanin polypeptide had no significant effect on cell activity and cisplatin injury. Therefore, the pathway of the direct effect of C-phycocyanin digestion was excluded. In addition to self-digestive enzymes, there are also intestinal microorganisms in the gut, which have been proved to be involved in the metabolism, immunity, and health of the host (34). At the same time, this *in vitro* experiment also proved that our previous therapeutic effect of C-phycocyanin on acute liver oxidative injury caused by X-ray needs the participation of gut microbiota (35). Therefore, we focus on the most important possibility of its role: gut microbiota and their metabolites.

Gut microbiome analysis showed that five bacterial genera are significantly related to cisplatin chemotherapy and C-phycocyanin prevention: *Lactobacillus, Romboutsia, Faecalibaculum, Bacteroides*, and *Enterococcus*. As a currently recognized probiotic genus, *Lactobacillus* has been confirmed by articles that *Lactobacillus* improves renal function and pathology in mouse models of AKI and chronic kidney disease (CKD) (36), which is consistent with our results. Network analysis showed that *Lactobacillus* is one of the top 10 abundant genera in our result, meanwhile, *Lactobacillus* is related to a large number of other intestinal microbial genera, which is consistent with the results of *Lactobacillus* ameliorates gut microbial dysbiosis in both AKI and CKD (36). *Lactobacillus* potently activated NRF2 in the drosophila and mice to improve oxidative damage and protect against oxidative liver injury (37), which is also confirmed in our result. Therefore, the activation of renal NRF2 anti-cisplatin oxidation pathway by C-phycocyanin is mediated by *Lactobacillus*, in particular, *Lactobacillus johnsonii* and *Lactobacillus reuteri*.

Reports showed that *Faecalibaculum* is always analyzed together with *Lactobacillus* because their abundance change is usually consistent (38, 39). Ingesting *Lactobacillus* in mice can also increase the abundance of *Faecalibaculum* (40), which means that the change of *Faecalibaculum* is regulated by *Lactobacillus*. Interestingly, although our results showed that the PC group increased the abundance of *Lactobacillus* and *Faecalibaculum* at the same time, there was no significant correlation between *Lactobacillus* and *Faecalibaculum*. We found that *Lactobacillus* only had a significant positive correlation with the relative abundance range of *Romboutsia ilealis* in the top 10 species. *Romboutsia ilealis* was first classified and separated by Gerritsen et al. (41). *Romboutsia ilealis* has limited ability to synthesize amino acids and vitamins, so it needs to be taken from the outside (42). C-phycocyanin is a protein rich in amino acids, so it can be used as a nutritional supplement to increase its abundance. However, how it works on the body and whether it is beneficial need to be further explored. It was also reported that the intervention of *Lactobacillus* could increase the abundance of *Romboutsia* (43); *Romboutsia* and *Faecalibaculum* also showed a significant positive correlation in our results. Therefore, we believe that there is a causal relationship between the three bacteria: the increased abundance of *Faecalibaculum* was achieved by increasing the abundance of *Romboutsia*, which was increased by *Lactobacillus*.

*Bacteroides* is different from *Lactobacillus* genius. Though there are pathogenic bacteria species in *Bacteroides*, such as *Bacteroides pyogenes* (44), yet probiotics like *Bacteroides acidifaciens*, which can guard against liver damages, are included. The other prebiotic effects of *Bacteroides acidifaciens* were summarized in our previous review (14). In our results, the abundance of *Bacteroides acidifaciens* in both the PC and CIS groups were increased, but compared with the CIS group, the abundance of *Bacteroides acidifaciens* in the PC group was decreased. *Bacteroides acidifaciens* has good consistency with liver injury. Combined with the literature (44), the real significance of *Bacteroides acidifaciens* bacteria may be used as a predictive biomarker of liver injury.

*Enterococcus faecalis* is a mammalian gut microbiota enriched in fatty acid-rich bile (45). *Enterococcus faecalis*, as a conditional pathogenic microorganism, changes into a pathogenic state when intestinal ecology is dysregulated, resulting in extensive infection (46). Recently, *Enterococcus faecalis* has been found to promote the migration and invasion of colon cancer cells (47). In our results, C-phycocyanin treatment decreased the abundance of *Enterococcus faecalis* caused by cisplatin chemotherapy, and the abundance of *Enterococcus faecalis* was significantly positively correlated with serum IL6 kidney MCP1 and liver IL1β levels, which means that the inflammatory damage caused by cisplatin chemotherapy is due to the increase of *Enterococcus faecalis*, and C-phycocyanin reduces the inflammation of cisplatin chemotherapy by reducing the abundance of *Enterococcus faecalis*.

The effect of gut microbiota on the host is produced by metabolites except for intestinal epithelial tissue (14). Through the analysis of high content metabolites significantly affected by C-phycocyanin and cisplatin, it was found that β-muricholic acid and 23-nordeoxycholic acid related to bile acid metabolism changed significantly. Cisplatin chemotherapy leads to the whole increase of muricholic acid, while the intervention of C-phycocyanin results to a significantly growing amount of 23-nordeoxycholic acid produced from the metabolism of β-muricholic acid. The high concentration of 23-nordeoxycholic acid is related to the inhibition of *Enterococcus faecalis*. Therefore, we believe that the inflammation induced by C-phycocyanin on cisplatin is realized through the 23-nordeoxycholic acid-*Enterococcus faecalis*-inflammatory factor axis.

Considering the close relationship between bile acid metabolism and fatty acids and their related metabolites, we focus on a new kind of small molecule substance FAHFAs related to nephritis (48). FAHFAs were originally classified by Yore et al. in 2014 and studied its mechanism in the body (49). They mainly found the important role of FAHFA (16:0, 18:0) in insulin resistance (49). FAHFA (18:2, 18:2) has also recently found its anti-inflammatory effect on body circulation (50). It is worth noting that although these beneficial FAHFAs can be completely absorbed into the blood by oral administration (51), the measurement of these beneficial FAHFAs is achieved in blood, in other words, they are endogenous lipids. The FAHFAs measured by fecal metabolomics do not match those beneficial FAHFAs, which also proves that these beneficial FAHFAs do not originate in the gut. In addition, although FAHFA (16:0, 18:0) and FAHFA (18:1, 18:0) play a positive role in diabetes, it also disrupts liver homeostasis in healthy mice (52). Therefore, we analyzed the intestinal-derived FAHFAs in detail. Interestingly, our fecal metabolome did not match these beneficial FAHFAs and found that the content of FAHFAs increased after cisplatin chemotherapy, and the content of FAHFAs decreased due to the intervention of C-phycocyanin, which was synchronized with the indexes of liver and kidney oxidation and inflammation, which means that fecal FAHFAs can be used as a trend marker of liver and kidney inflammation and oxidative damage. Because C-phycocyanin is extremely rich in amino acids, we also analyzed intestinal metabolites related to amino acid metabolism. N_α_-acetyl-arginine is the top drug candidate for mTORC1 inhibition (53). Derepresses mTORC1 signaling can initiate biliary-mediated liver regeneration (54). Combined with our results, the key intestinal substance for C-phycocyanin to improve liver injury might be N_α_-acetyl-arginine. Another important substance is trimethyl-lysine, which is an important post-translationally modified amino acid, and is linked to cancer, inflammation, and genetic disorders (55). The exact pathway of these two amino acid metabolites still needs to be further studied.

## Conclusion

C-phycocyanin taken orally exerted the preventive effect on cisplatin chemotherapy, especially reducing inflammation and enhancing the antioxidant capacity of the liver and kidney *via* gut microbiota and their metabolites. Microbial metabolite FAHFAs can be used as a predictor biomarker of renal injury and inflammation. *Enterococcus faecalis* is a biomarker bacteria of cisplatin chemotherapy inflammation and is regulated by 23-nordeoxycholic acid. The antioxidant effect of PC on the liver might be resisting Ferroptosis by GSH and its related enzymes and the antioxidant effect of C-phycocyanin on renal tissue is achieved by activating the NRF2 pathway. Increased gut metabolites by gavage C-phycocyanin and the activation of NRF2 is related to the rise of *Lactobacillus* genus.

## Data availability statement

The datasets presented in this study can be found in online repositories. The names of the repository/repositories and accession number(s) can be found below: https://www.ncbi.nlm.nih.gov/, PRJNA777291.

## Ethics statement

The animal study was reviewed and approved by Yantai Yuhuangding Hospital.

## Author contributions

LL designed the experiment. JY, XX, FW, and YZ carried out experiments. YZ analyzed the data. YZ, SQ, LL, and MC wrote the manuscript. YZ and SQ produced figures, table, and video. SH provided the experimental site. LL, YY, and SQ provided financial support. All authors contributed to the article and approved the submitted version.

## Funding

This work was supported by the Science and Technology Program of Yantai (2020MSGY076) and the Youth Innovation Promotion Association of the Chinese Academy of Sciences (2018246).

## Conflict of interest

The authors declare that the research was conducted in the absence of any commercial or financial relationships that could be construed as a potential conflict of interest.

## Publisher's note

All claims expressed in this article are solely those of the authors and do not necessarily represent those of their affiliated organizations, or those of the publisher, the editors and the reviewers. Any product that may be evaluated in this article, or claim that may be made by its manufacturer, is not guaranteed or endorsed by the publisher.

## Abbreviations

AKI: acute kidney injury
AREs: antioxidant response elements
CAS: Chemical Abstracts Service
CIS: cisplatin
CN: catalog number
CKD: chronic kidney disease
DMEM: Dulbecco's modified eagle medium
FAHFAs: fatty acid esters of hydroxy fatty acids
FBS: fetal bovine serum
GGT: γ-glutamyl transferase
GSH: glutathione
GSH-Px: glutathione peroxidase
i.p.: intraperitoneal injections
IL1β: interleukin 1β
IL6: serum interleukin 6
Keap1: Kelch-like ECH-associated protein 1
KEGG: Kyoto Encyclopedia of Genes and Genomes
LPS: lipopolysaccharide
MCP1: monocyte chemoattractant protein 1
mTORC1: mechanistic target of rapamycin complex 1
NRF2: NF-E2-related factor 2
PBS: phosphate buffered saline
PC: C-phycocyanin
SCFAs: short chain fatty acids
SOD: superoxide dismutase
WHO: World Health Organization.
